# Small biomarkers with massive impacts: PI3K/AKT/mTOR signalling and microRNA crosstalk regulate nasopharyngeal carcinoma

**DOI:** 10.1186/s40364-022-00397-x

**Published:** 2022-07-26

**Authors:** Hai-Long LI, Nian-Hua Deng, Xiu-Sheng He, Yue-Hua Li

**Affiliations:** 1 Key Laboratory of Cancer Cellular and Molecular Pathology in Hunan Province, Cancer Research Institute of Medical College, Hengyang Medical School, University of South China, 421001 Hengyang, Hunan P.R. China; 2 Key Lab for Arteriosclerology of Hunan Province, International Joint Laboratory for Arteriosclerotic Disease Research of Hunan Province, Institute of Cardiovascular Disease, Hengyang Medical School, University of South China, 421001 Hengyang, Hunan P.R. China; 3 Department of Medical Oncology, The First Affiliated Hospital, Hengyang Medical School, University of South China, 421001 Hengyang, P.R. China

**Keywords:** PI3K/AKT/mTOR, miRNAs, Nasopharyngeal carcinoma

## Abstract

Nasopharyngeal carcinoma (NPC) is one of the most common malignant tumours of the head and neck in Southeast Asia and southern China. The Phosphatidylinositol 3-kinase/protein kinase B (AKT)/mammalian target of rapamycin (mTOR) signalling pathway is involved in processes related to tumour initiation/progression, such as proliferation, apoptosis, metastasis, and drug resistance, and is closely related to the clinicopathological features of NPC. In addition, key genes involved in the PI3K/AKT/mTOR signalling pathway undergo many changes in NPC. More interestingly, a growing body of evidence suggests an interaction between this signalling pathway and microRNAs (miRNAs), a class of small noncoding RNAs. Therefore, in this review, we discuss the interactions between key components of the PI3K/AKT/mTOR signalling pathway and various miRNAs and their importance in NPC pathology and explore potential diagnostic biomarkers and therapeutic targets.

## Introduction

Nasopharyngeal carcinoma (NPC) is a malignant head and neck tumour that occurs in the top and sidewalls of the nasopharyngeal cavity [1]. NPC is uncommon compared to other tumour types and has a very unique geographic distribution, with more than 70% of new case reports occurring in East and Southeast Asia [2]. The incidence of NPC is related to Epstein–Barr virus (EBV) exposure, diet, and genetic factors [3]. Radiotherapy has achieved good results in the treatment of NPC because of its radiosensitivity [4, 5]. Approximately 95% of patients with early-stage nasopharyngeal carcinoma survive for more than 5 years, but only 54.2% of NPC patients are in the early stage [6]. In addition, NPC patients still suffer from locoregional recurrence, metastasis, and chemoradiotherapy resistance [7]. Therefore, further studies, particularly of early diagnostic biomarkers and radiation sensitization targets, are needed.

The phosphatidylinositol 3-kinase/protein kinase B (AKT)/mammalian target of rapamycin (mTOR) signalling pathway is vital to many hallmarks of tumours, such as cell growth, metabolism, and genomic instability, as well as angiogenesis and inflammation, and can function alone or in combination with many other important signalling pathways [8, 9]. Molecular studies have indicated that NPC pathogenesis involves multiple genetic and epigenetic alterations leading to uncontrolled activation of many signalling pathways, such as the PI3K/AKT/mTOR signalling pathway [10]. In line with this, a study indicated that abnormal activation of the AKT/mTOR pathway is related to the poor prognosis of NPC [11]. In addition, an increasing number of studies have shown that the abnormal activation of the PI3K/AKT/mTOR signalling pathway is involved in destroying the regulation of cell growth and survival, metastasis, and the development of radiation resistance [12, 13].

MicroRNAs (miRNAs) are noncoding 22–25-nucleotide-long RNA molecules that play important roles in regulating gene expression [14]. They bind to target mRNA molecules and specific proteins, thereby affecting their expression [15]. Many studies have shown aberrant expression of miRNAs in various human tumours, including NPC [16–18]. Moreover, many miRNAs have been demonstrated to be dysregulated in NPC [19, 20]. Previous studies have found that miRNAs regulate the expression of key genes via the PI3K signalling pathway in NPC and affect the proliferation, apoptosis, invasion, and metastasis of various tumours, including NPC [21, 22]. Interestingly, the PI3K/AKT/mTOR signalling cascade is also involved in miRNA biogenesis and biological function [23]. Therefore, there is great potential for regulatory miRNAs of the PI3K/AKT/mTOR signalling pathway as diagnostic biomarkers and radiation sensitization targets in NPC. In this review, we first discuss the PI3K/AKT/mTOR signalling pathway and its role in NPC. Furthermore, after a brief introduction to the biogenesis and functions of miRNAs, their potential clinical value in the diagnosis and treatment of NPC is discussed. Finally, the importance of the interaction between the PI3K/AKT/mTOR signalling pathway and tumour suppressor/oncogenic miRNAs in NPC is presented. This review comprehensively discusses the prospects of basic research on miRNAs and the PI3K/AKT/mTOR pathways for future clinical application in the treatment of NPC.

### The PI3K/AKT/mTOR signalling pathway: roles in cancers

**Fig. 1:**
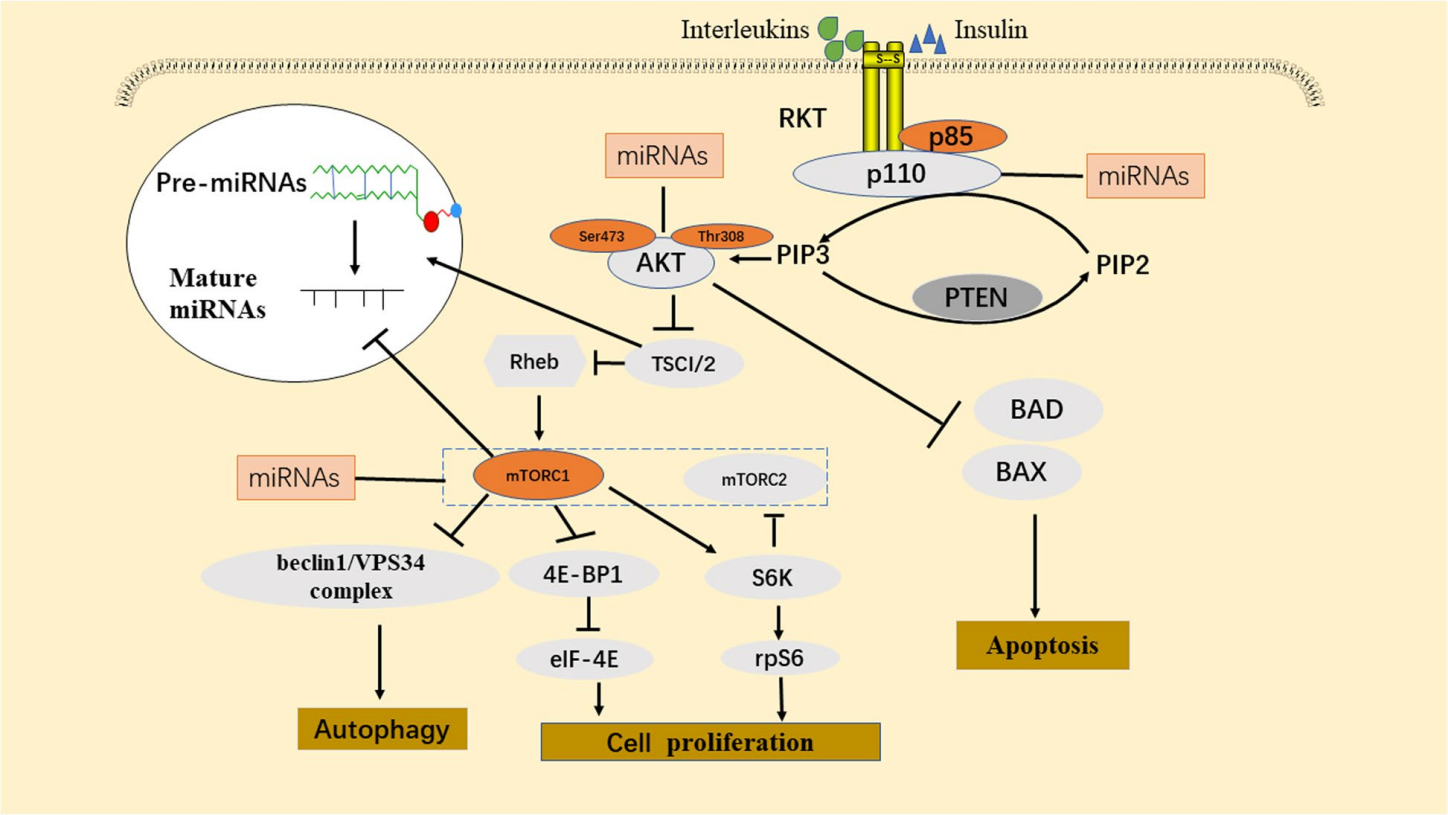
Interactions between miRNAs and key components of the PI3K/AKT/mTOR signalling pathway in cancers. External growth factors and miRNAs activate the PI3K/AKT/mTOR pathway, which directly and indirectly results in tumorigenesis, inhibition of apoptosis and autophagy, and activation of proliferation. AKT, protein kinase B; mTOR, mammalian target of rapamycin; PDK1, phosphoinositide-dependent kinase 1; PI3K, phosphoinositide 3‐kinase; 4E-BP1, eukaryotic translation initiation factor 4E binding protein 1; RPS6, ribosomal protein S6; eIF4E, eukaryotic translation initiation factor 4E; PIP3, phosphatidylinositol 3,4,5-trisphosphate; BAD, Bcl2-related death protein

The PI3K/AKT/mTOR signalling pathway plays an important role in fundamental cellular activities such as cellular metabolism, growth, and proliferation in many tumours [24] (Fig. 1). This signalling pathway can be activated by a variety of cellular stimuli, such as low levels of nutrients and/or oxygen supply, ionizing radiation, and pH [21]. Aberrant activation of PI3K signalling is frequently reported in aggressive tumours, such as NPC [25]. PI3Ks are lipid kinases that are divided into three different classes, including class I PI3Ks, class II PI3Ks, and class III PI3Ks [26]. Class I PI3Ks are heterodimers that consist of a p85 regulatory subunit and a p110 catalytic subunit (p110α, p110β, p110γ or p110δ). Class II PI3Ks are composed of a single catalytic subunit; PI3KC2α, PI3KC2β, and PI3KC2γ are three isoforms of class II PI3Ks that are stimulated by cytokine receptors, receptor tyrosine kinases (RTKs), and integrins. Class III PI3Ks are composed of a catalytic VPS34 subunit. Among them, class I PI3Ks are the most studied and have been implicated in tumorigenesis and tumour growth. The PIK3CA gene encoding the PI3K catalytic isoform p110α is considered to be one of the most frequently mutated oncogenes in many malignant tumours, including NPC [27]. The histidine residue (H1047) in the kinase domain and the acidic cluster (E542, E545, and Q546) in the helical domain are two hot spots containing PIK3CA gene mutations [28]. The serine/threonine kinase AKT is central to this pathway and consists of three distinct isoforms (AKT1-3) [29]. The mTOR kinase includes two distinct protein complexes: mTOR complex 1 and 2 (mTORC1 and mTORC2). Characteristically, mTORC2 is insensitive to rapamycin. Rapamycin is a potent inhibitor of mTOR complex 1 (mTORC1) signalling, and its activity is dependent on PI3K-mediated growth factor signalling [30, 31]. RTKs or G protein-coupled receptors (GPCRs) can activate PI3K, which senses interleukins, growth factors (such as insulin), and other external messengers. When PI3K is activated, it can catalyse the phosphorylation of PIP2 at position 3 of the inositol ring to produce PIP3 [32]. Phosphatase and tensin homologue (PTEN) negatively regulates this process [33]. PIP3 then recruits two protein kinases to the plasma membrane through its pleckstrin homology (PH) domains: AKT and phosphoinositide-dependent protein kinase 1 (PDK1). Once these two proteins are recruited to the cell membrane, AKT is phosphorylated by mTORC2 at the second residue (S473) and undergoes a conformational change that allows it to be phosphorylated on Thr308 by PDK1. In addition, the mTORC1 and mTORC2 complexes promote growth and enhance metabolism while weakening autophagy and apoptosis by phosphorylating many secondary molecules, such as ribosomal protein S6 kinase 1/2 (S6K1/2) [34, 35]. Activation of the PI3K/AKT signalling pathway promotes the activation of mTORC1. Subsequently, activated mTORC1 phosphorylates its downstream effectors, such as S6K and eukaryotic translation initiation factor 4E binding protein 1 (4E-BP1). Activated S6K phosphorylates ribosomal protein S6 (RPS6) and stimulates translation again. On the other hand, inactivated 4E-BP1 enhances the release of eukaryotic translation initiation factor 4E (eIF4E), which is regarded as an inhibitory factor in the process of translation initiation [36, 37]. It is worth noting that 4E-BP1 plays an important role in tumorigenesis, for example, promoting cell growth, protein translation, and drug resistance [38]. Therefore, this signal transduction plays an important role in tumours [39].

### The role of PI3K/AKT/mTOR signalling in NPC development and therapy

**Fig. 2:**
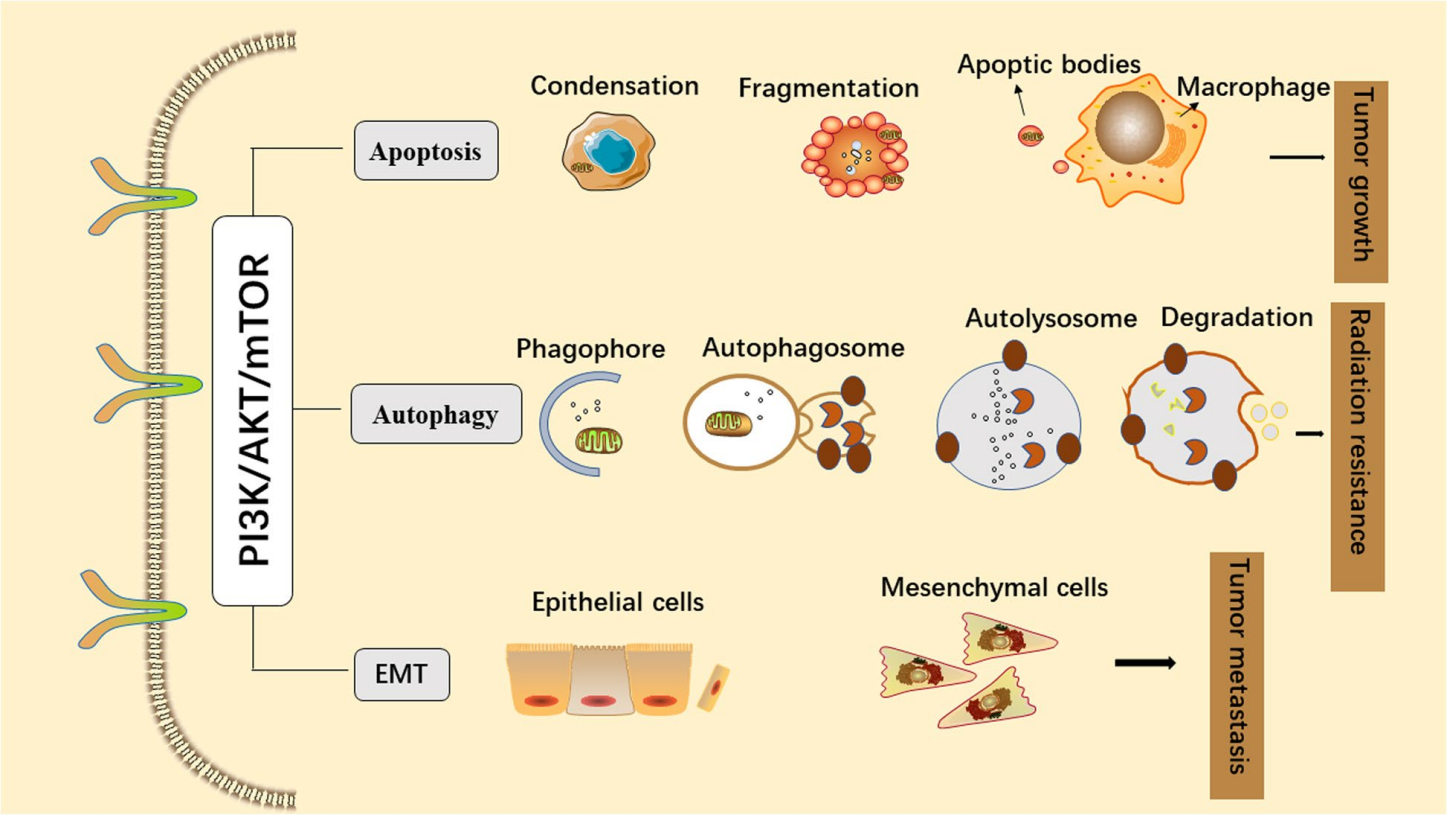
The underlying mechanisms of the PI3K/AKT/mTOR signalling pathway in NPC

Studies have found that since the PI3K/AKT/mTOR pathway is closely related to apoptosis, autophagy, and epithelial-mesenchymal transition (EMT), it can affect cell growth, proliferation, invasion, metastasis, and radioresistance in NPC [40–42] (Fig. 2). Cell growth is an important biological feature of organisms and is affected by the cell cycle and apoptosis. Numerous studies have indicated that the cell cycle is the convergence point of the PI3K/AKT/mTOR signalling cascade and that atypical cell cycle progression is an essential feature of tumours [43, 44]. The mTOR complex inhibitor rapamycin inhibits cell growth and the cell cycle [45]. Overexpression of constitutively active mutant S6K1 or eIF4E accelerates the G1 phase, indicating that 4E-BP1/eIF4E, as downstream signals of mTOR, regulate cell proliferation to a certain extent by controlling the cell cycle [46]. In addition, activated PI3K can directly inhibit tumour cell apoptosis [37]. The anti-apoptotic factor AKT can inhibit Bcl-2-associated death protein (BAD) and lead to the dissociation of Bcl-2 from the mitochondrial membrane, thereby inhibiting apoptosis [47]. Radiation resistance is a common phenomenon in NPC cells and is mainly related to autophagy [48]. The process of autophagy is broken down into four critical steps: initiation, nucleation, maturation, and degradation [49]. In various tumours, the activation of the PI3K/AKT/mTOR pathway promotes the initiation and nucleation of autophagy [50]. In the initial stage, foreign stimuli can activate the Unc-51-like kinase 1 (ULK1)-autophagy-related gene 13 (ATG13)-family interacting protein 200 kD (FIP200) kinase complex through the PI3K/AKT/mTOR pathway. During the nucleation step of autophagy, the ULK1 complex phosphorylates and activates the beclin1/PI3K III complex. The complex includes Beclin-1, PI3K III, and other proteins, such as VPS15 and ATG14L, depending on the subcellular localization of the complex. In addition, PI3K/AKT/mTOR signal transduction also plays a significant role in tumour metastasis via the induction of tumour EMT and angiogenesis [51]. For example, activated AKT upregulates key angiogenic factors, such as hypoxia-inducible factor-1 (HIF-1) and vascular endothelial growth factor (VEGF), to enhance tumour cell trafficking [52, 53].

Intriguingly, PI3K/AKT/mTOR signalling has been shown to be important in the treatment of NPC. The mainly treatment for NPC is radiotherapy and adjunct chemotherapy. A recent study showed that the PI3K/AKT/mTOR pathway is involved in not only radiotherapy resistance in NPC but also chemotherapy resistance [54]. Zhang et al. [55] found that PI3K/AKT signalling was involved in enhancing the radiosensitivity of NPC cells and reversing epithelial − mesenchymal transformation. Poor prognosis is a problem in the treatment of many tumours, including NPC. A study found that the prognosis of NPC patients with PI3K-AKT/mTOR signalling pathway mutations was poor. Next-generation sequencing of driver genes in the PI3K-AKT and mTOR signalling pathways is expected to provide new ideas for basic research and targeted therapy of NPC [56]. Another study showed substantial changes in PTEN, a key gene regulating the PI3K/AKT/mTOR pathway, after radical radiotherapy of NPC during long-term follow-up according to pathology and genomic phenotype assessment of secondary neuroendocrine carcinomas [57]. Therefore, it is very important to study the effect of PI3K/AKT/mTOR signalling on the prognosis of NPC. Furthermore, it has been found that the mechanism of many traditional Chinese medicines against NPC is regulation of the PI3K/AKT/mTOR pathway [58–60]. In general, the study of PI3K/AKT/mTOR signalling is of great significance for understanding the occurrence, development and treatment of NPC.

### miRNAs: clinical value in NPC

miRNAs, noncoding RNA molecules, play an essential role in posttranslational modification and protein synthesis [61]. Studies have indicated that these molecules can regulate more than 30% of the human genome [62, 63]. In addition, it has been reported that a large number of miRNAs are involved in the pathological process of NPC [64]. For example, miR-296-5p, miR-137, and miR-483-5p can regulate the migration and invasion of NPC cells [65–67]. Current research on miRNAs in tumours mainly uses miRNAs as diagnostic and prognostic biomarkers and therapeutic targets. Therefore, in this section, we will discuss the roles and clinical value of miRNAs in NPC.

### miRNAs as diagnostic and prognostic biomarkers

A large number of studies have shown that miRNAs can be used as diagnostic biomarkers of tumours, including NPC [68, 69]. Li et al. [70] found that the combination of three serum miRNAs, miR-29c-3p, miR-143-5p, and miR-205-5p, may be a new noninvasive biomarker for NPC screening. In addition, a model based on three miRNAs, miR-134-5p, miR-205-5p, and miR-409-3p, could be used as a marker for the diagnosis of NPC [71]. As a tumour suppressor, miR-29c is downregulated in the serum and tissues of patients with NPC, which indicates that it may be a molecular marker for the diagnosis of NPC [72]. In addition, a recent study identified circulating miR-31-5p as a potential new biomarker for the early diagnosis of NPC [73]. According to recent studies, increased expression of some miRNAs is associated with reduced overall survival and increased mortality of patients with NPC [74]. For example, the expression of miR-663 in the serum of patients with NPC was significantly higher than that in healthy people, and its expression was negatively correlated with the overall survival rate of patients with NPC [75, 76]. These results suggest that miR-663 can be used as a prognostic biomarker in NPC. Some studies have also shown that miR-342-3p, as a tumour suppressor, is an important molecular marker for the prognosis of patients with NPC [77]. These studies suggest that miRNAs can be used as potential diagnostic and prognostic biomarkers in NPC.

### miRNAs as radiation sensitization targets for NPC therapy

Enhancing the radiotherapy sensitivity of NPC is a topic that is being continuously explored by researchers [78]. Studies have detected differentially expressed miRNAs in radiosensitive and radioresistant NPC cells by gene sequencing and microarray analysis and found many differentially expressed miRNAs [79, 80]. Among them, miR-206 is downregulated in radioresistant NPC cells and enhances the radiosensitivity of NPC cells by targeting IGF-1 [81]. miR-23a was found to be involved in NPC radiotherapy resistance because it targets IL-8 [80]. Mechanistically, miRNAs first regulate the radiotherapy sensitivity of NPC cells by affecting the transmission of apoptosis-related signals. For example, miR-185 promotes radiotherapy sensitivity in NPC by regulating the Bcl-2 protein, an apoptosis suppressor [82]. In addition, miR-19b-3p has also been found to regulate Bcl-2 family proteins to inhibit radiotherapy sensitivity in NPC [83]. Second, miRNAs affect the sensitivity of NPC cells to radiotherapy by regulating DNA double-strand break repair. DNA repair in NPC cells after radiotherapy is mainly maintained by the telangiectasia mutated (ATM) and ataxia-telangiectasia mutated and Rad3-related (ATR) signalling pathways [84]. Zhou et al. [85] found that EBV-miR-BART8-3p could reduce the sensitivity of NPC cells to radiotherapy by regulating the activity of the ATM/ATR pathway. Furthermore, miRNAs can affect the radiosensitivity of NPC cells by regulating the cell cycle because cells with different cell cycle characteristics have different sensitivities to radiotherapy. A study found that miR-188 can lock NPC cells in the G1/S phase by inhibiting retinoblastoma protein (Rb) [86]. Moreover, another study found that miR-23a keeps cells in the G2-M phase by activating the IL-8/Stat3 pathway, thus sensitizing NPC cells to radiotherapy [87]. At present, many researchers are trying to find additional miRNAs that can function as potential radiotherapy sensitization targets for NPC [78]. For instance, miR-19b-3p was found to enhance radiotherapy resistance in NPC by activating the TNFAIP3/NF-κB axis [83]. Qu et al. [88] found that miR-205 is upregulated in radiotherapy-resistant NPC cells and can directly inhibit PTEN to increase radiotherapy resistance in NPC. miR-20a-5p can enhance the radiotherapy resistance of NPC cells by targeting the RAS oncogene family member Rab27B, which is associated with radiotherapy resistance of NPC and is also upregulated in radiotherapy-resistant NPC cell lines [89]. In addition, miR-193a-3p can attenuate the radiotherapy sensitivity of NPC cells by targeting the SRSF2 gene and hypoxia signalling pathways [90]. Therefore, enhancing the sensitivity of NPC cells to radiotherapy by targeting these miRNAs is a promising approach.

### Crosstalk between miRNAs and key components of the PI3K/AKT/mTOR signalling pathway

There are some interactions between miRNAs and the PI3K/AKT/mTOR signalling pathway (Fig. 1). For example, PI3K and its downstream components, such as AKT and mTOR, can be directly targeted by some miRNAs, and miRNA function can also be influenced by the PI3K/AKT signalling pathway. This interaction has critical roles in some cellular events, such as proliferation, apoptosis, and autophagy. Many miRNAs can either inhibit or activate the PI3K/AKT**/**mTOR signalling pathway by regulating its essential components. Regarding proliferation, Lv et al. [91] found that miR-520a-3p inhibited the proliferation of non-small-cell lung cancer through the PI3K/AKT/mTOR pathway. Sun et al. [92] found that miR-365 inhibits the PI3K/AKT pathway by targeting IGF-I, thereby inhibiting cell proliferation. In addition, it was found that miR-660-5p could promote breast cancer cell proliferation through the PI3K/AKT/mTOR pathway [93]. Therefore, miRNAs regulating the PI3K/AKT signalling pathway play an important role in regulating tumour cell proliferation. Regarding apoptosis, Jing et al. [94] found that miR-26a-5p regulates apoptosis by inhibiting the PI3K/AKT pathway in endothelial cells. Zhang et al. [95] found that miR-217 can inhibit apoptosis through the Toll-like receptor (TLR) 4/PI3K/AKT/NF-kB pathway in atherosclerotic endothelial cells. In addition, there are many miRNAs in NPC that affect the occurrence and development of tumours by inhibiting apoptosis. Zuo et al. [96] found that miR-155 inhibited the apoptosis of NPC cells through the PTEN-PI3K/AKT pathway. Therefore, an in-depth study of the mechanism by which mRNAs that regulate the PI3K/AKT signalling pathway regulate tumour cell apoptosis will provide a solid foundation for clinical applications. Regarding autophagy, many studies have shown that miRNAs play an important role in regulating PI3K/AKT/mTOR-mediated autophagy. Studies have shown that miRNA-mediated gene regulation can affect the AKT pathway, generating an AKT-miRNA regulatory network [97]. Gu et al. [98] found that miR-21 can inhibit autophagy by regulating the PI3K/AKT/mTOR pathway to regulate the resistance of gastric cancer cells to cisplatin. Meng et al. [99] found that miR-22 inhibits autophagy through the PI3K/AKT/mTOR pathway, thereby mediating cisplatin resistance in osteosarcoma. In addition, miR-21 also inhibits breast cancer cell autophagy through the PI3K/AKT/mTOR pathway and sensitivity to chemotherapeutic drugs [100]. Therefore, miRNAs that regulate the PI3K/AKT/mTOR signalling pathway play an important role in regulating the drug resistance of tumour cells. It was found that miR-338 can regulate the PI3K/AKT/mTOR pathway to inhibit autophagy in cervical cancer, suggesting that miR-338 can be used as a therapeutic target for cervical cancer [101]. Studies have found that many miRNAs can target PTEN to affect the PI3K pathway in cancer [102, 103]. miR-424-5p is a potential tumour suppressor gene that inhibits the development of breast cancer cells by regulating autophagy mediated by the PTEN/PI3K/AKT/mTOR pathway. miR-181 inhibits autophagy in non-small-cell lung cancer by promoting PTEN/PI3K/AKT/mTOR signalling to affect the occurrence and development of tumours [104]. However, Liao et al. [105] found that miR-381 can promote autophagy in prostate cancer cells by regulating the PI3K/AKT/mTOR signalling pathway. Therefore, miRNAs not only promote but also inhibit PI3K/AKT/mTOR-mediated autophagy in tumours.

The specific effects of this pathway on miRNA are not very clear. However, a study found that rapamycin, an mTOR inhibitor, significantly changed the miRNA expression profiles in cancer cells [106]. Furthermore, another study found that loss of tuberous sclerosis complex (TSC) leads to extensive suppression of the expression of precursor and mature miRNAs [107]. More interestingly, miRNA biogenesis can be increased by targeting mutated Raptor (an essential component of mTORC1) [108]. Studies have found that Drosha can mediate the ubiquitination of RNases, while mTOR can target and inhibit them [108]. These studies have revealed interactions between the PI3K/AKT/mTOR signalling pathway and miRNA biogenesis, though the field is still in its infancy.

Long noncoding RNAs (lncRNAs) are important noncoding RNAs that can indirectly regulate the PI3K/AKT/mTOR signalling pathway by targeting and adsorbing miRNAs. Some studies have found that tumour cells can regulate exosomal transfer of miRNA from fibroblasts by expressing lncRNAs, and the miRNAs can further regulate PI3K/AKT/mTOR signalling to affect the tumour microenvironment [109, 110]. In addition, other studies have found that lncRNAs can regulate PI3K/AKT/mTOR signalling through targeted adsorption of miRNAs, thereby affecting the growth and proliferation of a variety of tumour cells, including pharyngeal squamous cell carcinoma cells [111, 112]. However, there has been no report about lncRNAs regulating PI3K/AKT/mTOR signalling in NPC, and thus, the topic is worthy of exploration.

### Crosstalk between PI3K/AKT signalling and miRNAs in NPC pathological processes

The PI3K signalling pathway plays an important role in balancing cell survival and apoptosis to affect the pathological processes of NPC [113, 114]. At the same time, miRNAs have a substantial impact on the occurrence and development of NPC because they regulate the PI3K pathway [115]. Therefore, this section will explore the crosstalk between tumour suppressor and oncogenic miRNAs and the PI3K pathway in NPC (Table 1).

**Table 1:** miRNAs regulating the PI3K/AKT signalling pathway in the pathogenesis of NPC

miRNA	Target	Molecular alteration	Function	Reference
miR-3188	mTOR	Downregulation	Inhibits proliferation and chemoresistance	[116]
miR-331-3p	elF4B	Downregulation	Inhibits survival and metastasis	[117]
miR-34a	AXL	Downregulation	Inhibits invasion and EMT	[118]
miR-122	TRIM29	Downregulation	Inhibits progression and metastasis	[119, 120]
miR-375	USP1	Downregulation	Inhibits migration and invasion	[121]
miR-206	IGF1	Downregulation	Promotes radiosensitization	[81]
miR-29a	VEGF	Downregulation	Inhibits cell proliferation	[122]
miR-16	FGF2	Downregulation	Inhibits cell proliferation	[123]
miR-144-3p	PTEN	Upregulation	Promotes cell proliferation and invasion	[124]
miR-155	PTEN	Upregulation	Promotes proliferation and inhibits apoptosis	[96]
miR-205-5p	PTEN	Upregulation	Promotes the EMT	[125]
miR-144	PTEN	Upregulation	Promotes cell proliferation and migration	[126]
EBV-miR-BART7-3p	PTEN	Upregulation	Enhances cell migration and invasion	[127]
EBV-miR-BART1	PTEN	Upregulation	Enhances cell migration and invasion	[127]
miR-192	RB1	Upregulation	Induces cell growth, invasion, and metastasis	[128]
miR-93	TGFβR2	Upregulation	Inhibits apoptosis	[129]

### Tumour suppressor miRNAs and PI3K/AKT signalling

Studies have shown that tumour suppressor miRNAs regulating the PI3K/AKT signalling pathway have a substantial impact on the growth, apoptosis, metastasis, and drug resistance of NPC cells [116, 118]. It was found that miR-3188 was downregulated in head and neck tumours, non-small-cell lung cancer, breast cancer, and liver cancer and inhibited tumour cell growth [130–132]. Recently, miR-3188 was identified as a target of mTOR signalling that can inhibit the proliferation and chemoresistance of NPC cells by targeting the mTOR-PI3K/AKT-c-JUN signalling pathway [116]. In addition, it has been reported that miR-331-3p downregulation in NPC cells is related to increased tumour cell survival and metastasis. Mechanistically, upregulation of miR-331-3p induced cell apoptosis while preventing cancer cell invasion by targeting the elF4B gene and then inhibiting the PI3K/AKT signalling pathway [117]. Consistently, Ma et al. [133] found that miR-34a promoted cell proliferation and inhibited apoptosis in papillary thyroid carcinoma through the PI3K/Akt/Bad pathway. However, Jiang et al. [118] found that miR-34a inhibited cell invasion and EMT by targeting AXL/PI3K/AKT/Snail signalling in NPC. Furthermore, there was a negative association between miR-122 expression and NPC growth. The expression of miR-122 was considerably suppressed in NPC cells. Upregulated expression of miR-122 led to reduced tumour progression and metastasis by downregulating TRIM29 and blocking PI3K/AKT signalling [119, 120]. A study indicated that TRIM29 upregulated PI3K/AKT signalling by reducing PTEN expression and increasing the levels of phosphorylated AKT, p70S6K, and 4E-BP1 [134]. According to the study, miR-375 effectively repressed colorectal cancer development by targeting the PI3K/AKT signalling pathway [121]. Upregulated miR-375 expression led to USP1 downregulation, and miR-375 overexpression inhibited NPC cell migration and invasion by suppressing PI3K/AKT signalling [135]. The study also found that miR-206 directly targets IGF-1, a PI3K/AKT pathway activator, and promotes NPC radio-sensitization [81]. Therefore, miR-206 is expected to be a target for radiotherapy sensitization in NPC. According to current studies [136, 137], miR-29a has both inhibiting and promoting effects in tumours, including cervical cancer and breast cancer. However, Shi et al. [122] found that high expression of miR-29a in NPC cells inhibited cell growth and increased apoptosis. Mechanistically, miR-29a targets VEGF and inhibits the activation of the PI3K/AKT and JAK/STAT pathways. miR-16 has been identified as a tumour suppressor gene, and its main role is to induce apoptosis by targeting Bcl-2 [138]. He et al. [123] aimed to further explore the mechanism of miR-16 in NPC and found that miR-16 inhibited the growth of NPC cells via the PI3K/AKT pathway by directly targeting fibroblast growth factor 2 (FGF2). Together, these results indicate that suppressor miRNAs that regulate the PI3K/AKT pathway suppress NPC carcinogenesis and progression, thereby representing potential targets for miRNA-based therapy for NPC.

### Oncogenic miRNAs and PI3K/AKT signalling

PTEN is a potent tumour suppressor and contributes to the regulation of cell survival, apoptosis, proliferation, metabolism, and migration by suppressing oncogenic PI3K signalling [139]. Moreover, many studies have indicated that negative regulation of PTEN is related to cancer progression in NPC patients [140, 141]. Furthermore, PTEN was identified as a potential target for oncogenic miRNAs in patients with NPC. For instance, miR-144-3p, miR-155, miR-205-5p, and miR-144, oncogenic miRNAs that are overexpressed in some cancers, induce NPC cell invasion, migration, and proliferation but restrain apoptosis directly by targeting the PTEN tumour suppressor, leading to upregulation of PI3K/AKT signalling [96, 124–126]. Studies have found that miR-144-3p is significantly overexpressed in NPC tissues and can enhance the proliferation and migration of NPC cells by targeting PTEN [124]. Zuo et al. [96] found that high expression of miR-155 in NPC cells promotes proliferation and inhibit apoptosis by targeting the PTEN/PI3K/AKT pathway. Some studies have found that miR-144 can inhibit PTEN signalling and promote cell proliferation and migration [126, 142]. Zhang et al. [125] found that miR-205-5p can promote the EMT of cisplatin-resistant NPC cells through the PI3K/AKT pathway and target PTEN. NPC is an Epstein–Barr virus (EBV)-associated malignancy with characteristic early metastasis. Consistently, Cai et al. [127, 143] indicated that EBV-miR-BART7-3p and EBV-miR-BART1, which are highly expressed in NPC, enhance NPC cell migration and invasion directly by targeting PTEN to modulate PI3K/AKT signalling.

In another study, Huang et al. [128] indicated that miR-192 significantly induces PI3K/AKT signalling by suppressing RB1 protein expression in NPC cells. Lyu et al. [129] showed that upregulation of the oncogenic miRNA miR-93 induces NPC cell growth, invasion, metastasis, and EMT-like processes by suppressing TGFβR2 by promoting the PI3K/AKT pathway. Additionally, Yang et al. [144] found that EBV-encoded LMP1 upregulates miR-21 to increase the resistance of NPC cells to cisplatin-induced apoptosis by inhibiting PDCD4 and Fas-L activity through the PI3K/AKT/FOXO3a pathway.

## Conclusion and perspective

NPCs mostly occur in Southeast Asia and have a high degree of malignancy; thus, they seriously endanger people’s lives and health [145]. The guidelines recommend radiotherapy and nonspecific cytostatic drugs, which seriously reduce the quality of life of patients and incur massive treatment costs. In addition, radiation resistance and chemotherapy resistance lead to unsatisfactory treatment effects in some patients [146]. Due to the prominent role of the PI3K/AKT/mTOR pathway in cell proliferation and survival, inhibitors of this pathway are anticipated to be effective treatments for NPC [147]. Preclinical trials showed that PI3K inhibitors decreased cell proliferation, decreased xenograft tumour growth, and increased radiosensitivity [148]. In addition, Liu et al. [149] found that the dual PI3K/mTOR inhibitors GSK216458 and PKI-587 inhibited the growth of NPC cells and enhanced their radiosensitivity. Radiotherapy combined with dual PI3K/mTOR inhibitors may be a promising treatment strategy for NPC. Preclinical evaluation showed that the mTOR-PI3K inhibitor BEZ235 caused G1 arrest and increased apoptosis in most NPC cell lines [150]. Regarding AKT inhibitors, the preclinical results of studies in which such inhibitors are combined with radiotherapy and administered to cells with abnormally elevated levels of p-Akt and P-S6 kinase are very promising [151]. For ethical reasons, AKT inhibitors are currently only used in very advanced patients. Furthermore, another study found that Rad001, an mTOR inhibitor, had a synergistic effect on cisplatin-induced growth inhibition of NPC cells and inhibited the growth of cisplatin-resistant and cisplatin-sensitive NPC cell lines [152]. These results suggest that mTOR inhibitors combined with cisplatin may be an effective treatment strategy for NPC. Future trials should focus on combining radiotherapy with a variety of targeted PI3K/AKT/mTOR pathway inhibitors to take advantage of possible synergistic effects and investigate whether patients with radiation resistance can also benefit from these combinations.

Increasing evidence indicates the significance of miRNAs in the regulation of the PI3K/AKT/mTOR signalling pathway in NPC. The PI3K/AKT/mTOR signalling pathway is activated by enzyme-linked receptors, which have substantial effects on tumour cell proliferation, apoptosis, and autophagy. These effects cause diverse outcomes, creating the complex characteristics of NPC. Abnormal changes in this pathway may underly radiotherapy resistance in NPC. Multiple miRNAs can inhibit or promote pathway activation in the same manner as external molecules. Interestingly, there are many dysregulated miRNAs in NPC cells that have extremely strict effects on the PI3K/AKT/mTOR pathway. In this review, many miRNAs regulating the PI3K/AKT/mTOR pathway were presented, and their importance in NPC pathology was discussed. In summary, the roles of miRNA-PI3K interactions in NPC were highlighted in this review, and novel strategies for NPC diagnosis and therapy were presented. Currently, miRNAs have promise in clinical applications, for example, the application of miRNAs as diagnostic biomarkers and the application of miRNA-based drugs that inhibit oncogenic miRNAs or promote tumour suppressor miRNAs. In addition, further study of the interaction between miRNAs and the PI3K pathway and how it affects the occurrence and development of NPC will accelerate the clinical application of strategies related to regulatory miRNAs. Therefore, future studies may focus on (1) identifying the best miRNA candidates for NPC diagnosis and treatment, (2) identifying the mechanisms underlying miRNA-PI3K interactions, and (3) developing new miRNA-based treatment strategies to control pathological response and better manage NPC.

In addition, many researchers have a strong interest in reversing dysregulation of miRNAs regulating the PI3K/AKT/mTOR pathway in NPC. Strategies related to exosomes may be useful [153]. Exosomes are small biological vesicles that can carry miRNAs, DNA, metabolites and small molecule drugs [154, 155]. They are very important for information transmission between cells. Studies have shown that exosomes have cell selectivity and tissue specificity and can accurately transport “goods” from parental cells to recipient cells [156, 157]. In the future, researchers may be able to load miRNAs that enhance the radiotherapy sensitivity of NPC cells into exosomes so that NPC cells can uptake these miRNAs and overexpress them, enhancing therapeutic effects in patients with radiation resistance. However, the technologies needed for the separation, purification and drug loading of specific exosomes do not yet exist and thus are worthy of further discussion.

## Data Availability

Not applicable.

## Abbreviations

PI3K: Phosphatidylinositol 3-kinase
AKT: Protein kinase B
mTOR: Mammalian target of rapamycin
miRNAs: MicroRNAs
lncRNAs: Long noncoding RNAs
NPC: Nasopharyngeal carcinoma
EBV: Epstein–Barr virus
PDK1: Phosphoinositide-dependent protein kinase 1
4E-BP1: 4E binding protein 1
eIF4E: Eukaryotic translation initiation factor 4E
EMT: Epithelial-mesenchymal transition
BAD: cl-2-associated death protein
ULK1: Unc-51-like kinase 1
HIF-1: Hypoxia-inducible factor-1
TLR: Toll-like receptor
TSC: Tuberous sclerosis complex
FGF2: Fibroblast growth factor 2
